# Expression of Programmed Death Ligand-2 is associated with Prognosis in Nasopharyngeal Carcinoma Microenviroment

**DOI:** 10.7150/jca.77643

**Published:** 2022-11-14

**Authors:** Aixin Li, Weijun Wu, Shengling Deng, Qiao Yang, Junyan He, Haibiao Wu, Haiyun Wang, Jiaxing Zhang, Qisheng Feng, Jianyong Shao, Yixin Zeng, Manbo Cai

**Affiliations:** 1 Department of Radiotherapy, the First Affiliated Hospital, Hengyang Medical School, University of South China, Hengyang, Hunan, 421001, China.; 2 Department of Anesthesia, the Second Affiliated Hospital, Hengyang Medical School, University of South China, Hengyang, Hunan, 421001, China.; 3 State Key Laboratory of Oncology in South China, Cancer Center, Sun Yat-Sen University, Guangzhou, 510060, China.

## Abstract

**Background:** Although immune checkpoint inhibitors have opened a new mode of treatment for solid tumors, their efficacy in nasopharyngeal carcinoma (NPC) needs to be further investigated. Inhibitors of the PD-1/PD-L1 immune checkpoint are one of the hot topics in tumor immunotherapy. Programmed death ligand-2 (PD-L2) is a less studied ligand of PD-1 and has not yet been fully explored, especially in NPC. Understanding the clinical significance of PD-L2 expression, together with immune cell infiltration, might provide clues for biomarker screening in NPC immunotherapy. This study aimed to evaluate the role of PD-L2 as a prognostic factor for NPC patients as well as its role in immune regulation.

**Methods:** Immunohistochemistry (IHC) was performed on a tissue microarray including 557 NPC specimens using PD-L2 antibody. The immune cell markers CD4, FOXP3 and CD68 were also stained and quantified. The expression of PD-L2 exhibited different spatial patterns among NPC tumor and stromal tissues.

**Results:** A total of 90.8% of the cases showed membranous PD-L2 expression in tumors, and 80.8% showed membranous PD-L2 expression in stromal tissue. High stromal expression of PD-L2 predicted favorable overall and disease-free survival of NPC patients and was negatively correlated with tumor size, recurrence or metastasis and clinical stage. In contrast, high tumor abundance of PD-L2 correlated with poor disease-free survival, but had no obvious correlation with clinicopathological parameters. Multivariate analysis indicated that stromal PD-L2 was an independent and favorable prognostic factor. Furthermore, we found a positive correlation between stromal PD-L2 expression and the infiltration of CD68^+^ macrophages and CD4^+^Foxp3^+^ Treg cells in NPC stromal tissues (Pearson correlation=0.181 and 0.098, respectively).

**Conclusions:** Our results suggest that different PD-L2 expression patterns have distinct predictive values. PD-L2 expressed on stromal cells might play a role in the regulation of NPC progression, and involve in immune activation in the tissue microenvironment and have an independent good prognosis for NPC patients.

## Introduction

NPC is one of the most common head and neck malignancies and is highly prevalent in Southeast Asia, North Africa and southern China 1. It is characterized by Epstein-Barr virus (EBV) infection and heavy lymphocyte infiltration 2. Seventy percent of NPC patients have locally advanced disease at the first diagnosis. The wide use of intensity-modulated radiotherapy and the optimization of chemotherapy strategies have dramatically improved treatment outcomes and overall prognosis over the past few decades 3. However, they have a limited effect on patients with locally advanced or distantly metastatic disease, and approximately 30% of high-risk patients still experience tumor recurrence, with distant metastasis as the primary failure pattern 4.

Nevertheless, the unique immune environment of EBV-associated NPC indicates the potential benefits of immunotherapy based on immune checkpoint inhibitors (ICIs). Programmed cell death-1 (PD-1) is mainly expressed on activated T cells and acts as an immune activation reaction of tumors after the combination of inhibitory receptor and ligand (PD-L1). In recent years, the application of inhibitors targeting PD-1 and PD-L1 in solid tumors has made substantial progress. In several clinical trials exploring the role of PD-1/PD-L1 inhibitors in NPC, Fang W, Ma B and Hsu C showed a good objective response rate with immune checkpoint inhibitors in patients with recurrent or metastatic NPC 5-7. Furthermore, in Fang et al.'s report, camrelizumab (anti-PD-1 antibody) monotherapy combined with chemotherapy has made promising progress in the first-line treatment of recurrent and metastatic NPC 5. However, anti-PD-1therapies are effective in only 20~30% of NPC patients 5,7. Moreover, following the administration of immune checkpoint inhibitors, an unexpected pattern of response designated hyperprogression may be observed in certain patients, ranging between 4 and 29% 8. These results indicated that the tumor microenvironment is intricate and that other immune checkpoints might exist. Understanding the clinical significance of the ICI-associated tumor microenvironment might provide clues for biomarker screening in NPC immunotherapy.

PD-1 has two ligands: PD-L1 and programmed death ligand-2 (PD-L2). PD-L1 is the main ligand for PD-1 and is one of the major targets for cancer immunotherapy. Multiple studies are available on the clinical significance of PD-1/PD-L1 in NPC, and the prognostic value remains controversial. Meta-analysis and IHC analysis showed that PD-L1 overexpression in NPC was associated with poor overall survival (OS) 9-10. Some retrospective analyses found that PD-L1 expression in immune cells is a favorable prognostic factor for NPC 11. However, another meta-analysis revealed that PD-L1 expression in NPC did not correlate with OS, and no statistically significant differences existed between the expression level of PD-1 in tumor infiltrating lymphocytes (TILs), which indicated that higher/positive expression of PD-L1/PD-1 may not serve as suitable biomarkers for the prognosis of NPC 12. In comparison to PD-L1, PD-L2 is a less studied ligand of PD-1. Some scholars believe that PD-L2 may be a potential therapeutic response marker of head and neck squamous cell carcinoma 13-14. However, to date, the expression and role of PD-L2 in NPC have yet to be fully explored.

Here, to explore the role of PD-L2 in the NPC tumor microenvironment, we first examined PD-L2 expression in NPC tumor cells and stromal cells using the IHC method. Second, we analyzed the correlation between the PD-L2 expression level and the clinical factors and outcomes of NPC patients. Finally, we evaluated the possible immune-regulatory function of PD-L2 on CD68^+^ macrophages and CD4^+^Foxp3^+^ Treg cells in the tumor microenvironment in NPC.

## Patients and methods

### Patients and clinical tissue samples

In this study, 557 NPC specimens were collected at the Department of Pathology, Sun Yat-Sen University Cancer Center (SYSUCC), Guangzhou, China, between January 2001 and December 2003. The inclusion criteria of patients and follow-up were the same as those in previous publications 15. All patients were treated with standard curative radiotherapy, with or without chemotherapy. The Institute Research Medical Ethics Committee of Sun Yat-Sen University granted approval for this study.

### Tissue microarray construction

Paraffin-embedded specimens were obtained from a previously constructed tissue microarray (TMA) 16. Briefly, the paraffin-embedded tissue blocks and the corresponding hematoxylin and eosin (H&E)-stained slides were overlaid for TMA sampling. Duplicates of 1.0 mm diameter cylinders were punched from representative tumor areas of individual tissue blocks and re-embedded into a recipient paraffin block at a defined position using a tissue arraying instrument (Beecher Instruments, Silver Spring, MD).

### IHC and evaluation

IHC was performed to examine PD-L2, CD68, CD4 and Foxp3 expression in NPC tissue specimens. Primary antibodies against PD-L2 (1:500 dilution, ab214221, Abcam, USA), CD4 (1:500 dilution, ab183685, Abcam, USA), Foxp3 (1:200 dilution, AF3240, USA), and CD68 (1:500 dilution, ABIN370601, Dako, Denmark) were used in this study. Formalin-fixed paraffin-embedded tissue specimens were processed in tissue microarrays with cores of 2 mm diameter. CD4^+^ antibodies were used to identify T lymphocytes (TIL, red membranous staining), and FoxP3 antibodies were used to identify regulatory T cells (Treg, brown nuclear staining). Immunohistochemistry was performed as double staining using a CD4-specific antibody and a FoxP3-specific antibody. For detection, a Polymer-Kit and Fast Red and Polymer-Kit and Fast brown (EnVision +Dual Link Kit) were used according to the manufacturer's instructions. The IHC results were evaluated and scored as described previously 16. In addition, the numbers of CD68^+^ macrophages and CD4+Foxp3+ Treg cells in the tumor and stroma were counted within the areas of 6 high-power fields (400X).

### Selection of cutoff score

Receiver operating characteristic (ROC) curve analysis was performed to determine the cutoff score for a “high expression” designation with the 0,1-criterion implemented in SPSS software 16. First, the clinicopathologic characteristics were dichotomized into the following groups: T classification (T1-T2 versus T3-T4), N classification (N0 versus N1-N3), clinical stage (I-II versus III-IV), cancer progression (Yes versus No) and survival status (death due to NPC versus censored). Second, the expression scores for PD-L2 were trained in the ROC analysis. The cutoff score is the point on the curve that has both maximum sensitivity and specificity 16-17.

### Statistical analysis

Statistical analysis was performed using SPSS (version 23.0; SPSS, Chicago, IL). The survival time was calculated monthly, and the OS time was calculated from the date of initial diagnosis to the date of death or the last follow-up. The χ^2^ test or Fisher's exact probability method was used for comparisons between groups. The survival rate was analyzed by Kaplan-Meier and log-rank methods. The Cox regression model was used for multivariate analysis. Associations between PD-L2 expression and the number of CD68^+^ macrophages or CD4^+^Foxp3^+^ Treg cells were examined by using Pearson correlation and independent t tests. P<0.05 was regarded as statistically significant.

## Results

### Patient characteristics

Among the 557 patients, 415 (74.5%) were male, and the median age was 46 years (range 19-78 years). Among these patients, 139 patients (25.0%) were diagnosed with nonkeratinizing differentiated carcinoma (NKDC), and 402 patients (72.2%) were diagnosed with nonkeratinizing undifferentiated carcinoma (NKUC), 16 patients (2.8%) were diagnosed with keratinizing squamous cell carcinoma (KSCC). One hundred forty-seven patients (26.4%) were in stage I and stage II, and 410 patients (73.6%) were in stage III and stage IV. The median follow-up time was 58.63 months (range 2 to 99 months). Of all the patients, 184 (33.0%) died, and 208(37.3%) presented with disease progression during the five-year follow-up period. Detailed clinical information is shown in supplemental Table 1.

### Expression of PD-L2 in NPC tumor tissues

**Figure 1:**
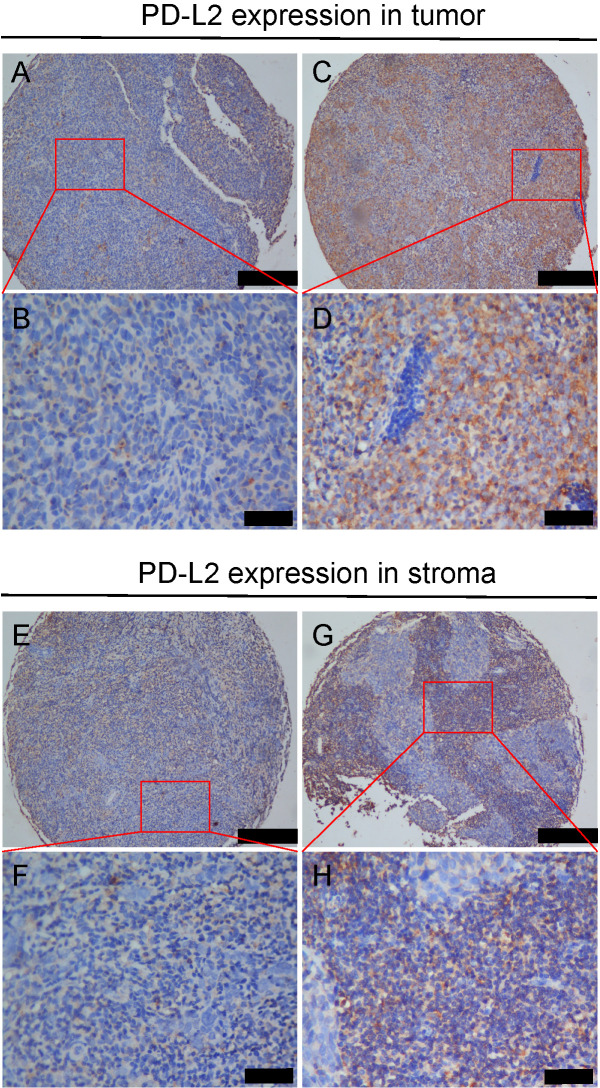
** Expression of PD-L2 in NPC tumor and stoma tissues. A and B** Weak PD-L2 staining in NPC tumor tissues of TMA samples under low and high magnifications; **C and D** Strong PD-L2 staining in tumor tissues is shown under low and high magnifications; **E and F** Weak PD-L2 staining in NPC stoma tissues of TMA samples under low and high magnifications; **G and H** Strong PD-L2 staining in stroma tissues is shown under low and high magnifications.

The expression of PD-L2 protein was determined in 557 NPC tumors from which five-year follow-up information was available by using IHC with the specific antibody. Overexpression of PD-L2 was observed in the membrane of NPC cells with different intensities (Figure 1). A total of 90.8% of the cases showed membranous PD-L2 expression in NPC tumor tissue (Figure 1A-D) and 80.8% of the cases showed membranous PD-L2 expression in stromal tissue (Figure 1E-H). Immunoreactivity for PD-L2 was scored for subsequent evaluation of its predictive value in NPC.

### Selection of the cutoff score for high expression of PD-L2

**Figure 2:**
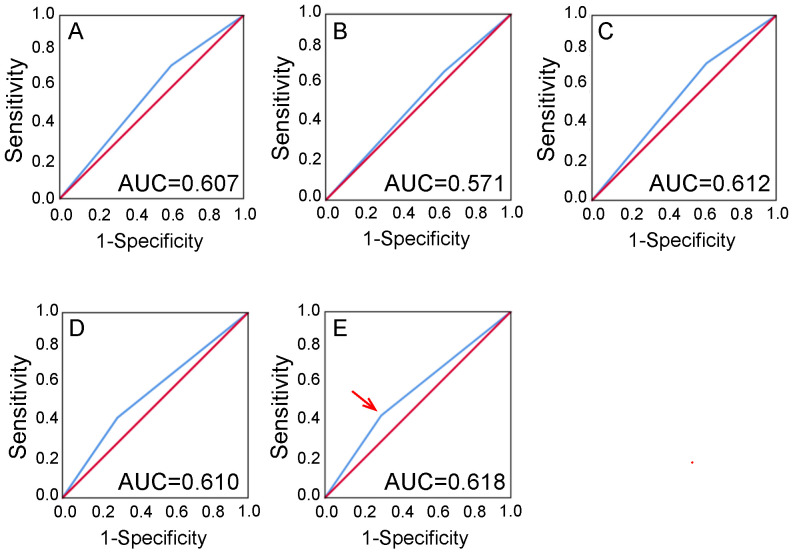
** Selection of the cutoff score.** Receiver operating characteristic curve analysis was employed to determine the cutoff score for the designation of “high expression” of PD-L2. The sensitivity and specificity for each outcome of PD-L2 membranous staining of stromal tissues were plotted: **A** T classification (AUC=0.607, P =0.001); **B** N classification (AUC=0.571, P =0.120); **C** Clinical stage (AUC=0.612, P =0.002); **D** Cancer progression (AUC=0.610, P=0.001); **E** Survival status (AUC=0.618, P=0.001).

ROC curve analysis showed that stromal PD-L2 expression has some sort of predictive value in NPC, with the maximum area under the curve (AUC) reaching 0.618 (Figure 2E, red arrow). The points in curves with both maximum specificity and maximum sensitivity were treated as cutoff points for high or low stromal expression of PD-L2. For survival analysis, the cutoff score for stromal expression was 3.5 (Table 1). Thus, tumors designated as low expression of PD-L2 are those with scores below or equal to the cutoff score, while stroma cells of high expression are those with scores above the cutoff score. Using the same method, we obtained a cutoff value of 5.5 for PD-L2 in tumor tissues (Table 1 and supplemental Figure 1).

### Association of PD-L2 expression with clinicopathologic features and survival

NPC samples were separated into either low or high PD-L2 expression groups in tumor and stromal tissues respectively according to their expression levels to the cutoff. As a result, high stromal expression of PD-L2 was observed in 364/557 (65.4%) NPC tissues. With χ^2^-test analysis, we found that high stromal expression of PD-L2 was negatively correlated with age (Pearson's R=-0.108, P=0.011), tumor T classification (Pearson's R=-0.123, P=0.004), recurrence and metastasis (Pearson's R=-0.127 P=0.003) and clinical stage (Pearson's R=-0.111 P=0.009), respectively (Table 2), while there was no significant association with other clinicopathologic features, such as patient sex, N classification and histological type (*P*>0.05, Table 2). Among the 557 patients, non-keratinizing carcinoma accounted for 97.2% (including 402 NKUC and 139 NKDC) (supplemental Table 1). Therefore, only the correlation between PD-L2 expression and pathological types of 541 non-keratinizing carcinoma was analyzed. On the other hand, high tumor expression of PD-L2 was observed in 127/557 (22.8%) NPC tissues. Furthermore, we found that high tumor expression of PD-L2 was positively correlated with histological type (Pearson's R=0.171 *P*<0.001), but there was no significant association with other clinicopathologic features, such as age, sex, T classification, N classification, recurrence and metastasis and clinical stage (*P*>0.05, Table 3).

**Figure 3:**
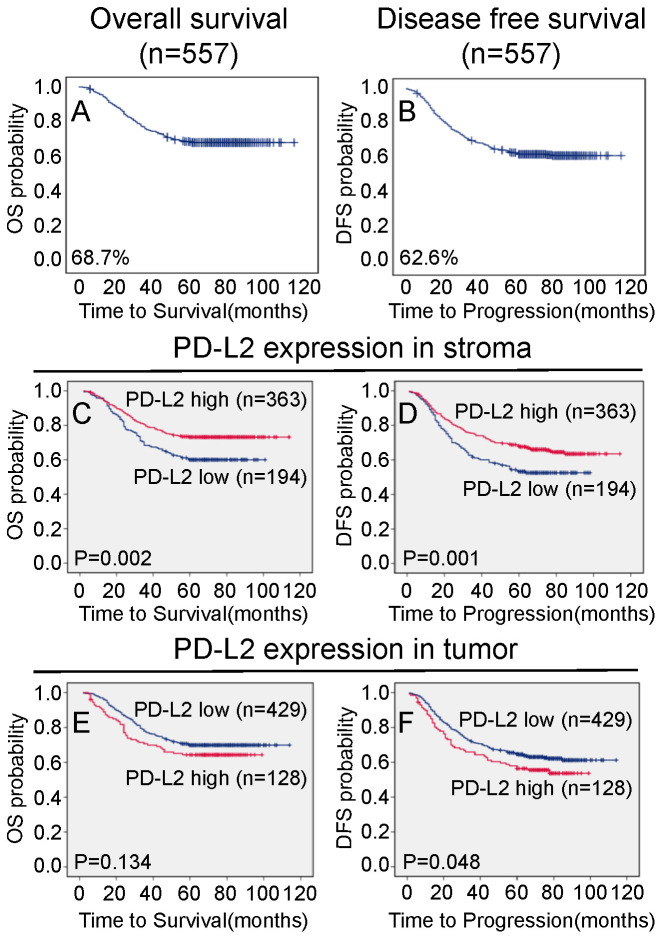
** Association between PD-L2 expression and NPC patient survival.** TMA analyses were conducted in a cohort of 557 NPC patients diagnosed at M0. **A** The five-year overall survival (OS) rate was 68.7%. **B** The five-year disease-free survival (DFS) rate was 62.6%. **C and D** High PD-L2 expression levels in stromal tissues were significantly associated with OS (P = 0.002) and disease-free survival (P = 0.001) in all NPC patients. **F** High PD-L2 expression levels in tumor tissues were significantly associated with disease-free survival (P = 0.048), and there were no significant differences in five-year OS (P = 0.134) in all NPC patients **(E)**.

**Figure 4:**
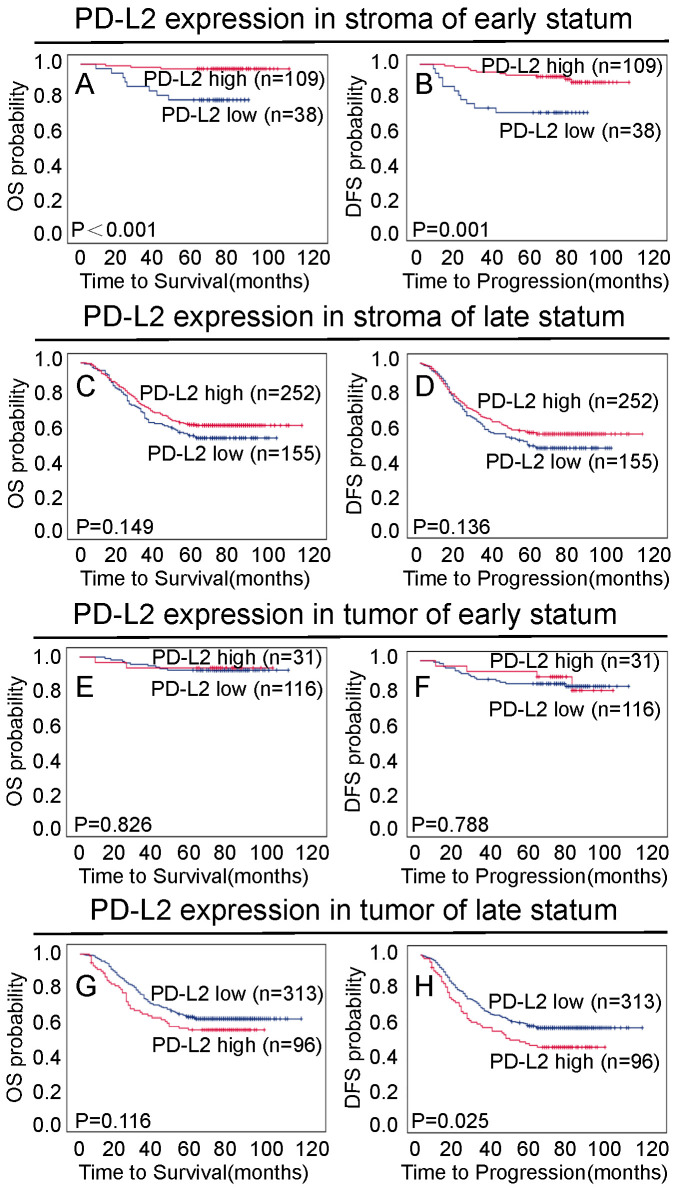
** Association between PD-L2 expression and NPC patient survival at different stages. A and B** High PD-L2 expression in the stroma was significantly associated with OS (P < 0.001) and disease-free survival (P = 0.001) in NPC patients at early stages (I-II). **C and D** No significant differences in five-year OS and DFS rates were found between groups with low and high expression of PD-L2 in tumor tissues of NPC patients at early stages (I-II). **E and F** No significant differences in five-year OS and DFS rates were found between groups with low and high expression of PD-L2 in NPC patients at late stages (III-IV).** H** High PD-L2 expression levels in tumor tissues were significantly associated with disease-free survival (P = 0.025), and there were no significant differences in five-year OS in all NPC patients **(G)**.

The 5-year overall and disease-free survival rates of the cohort of 557 NPC patients were 68.7% and 62.6%, respectively (Figure 3A and 3B). Of these 557 NPC patients, Kaplan-Meier and log-rank test analyses indicated that high stromal PD-L2 expression was significantly associated with favorable overall survival (OS) (5-year survival rates, 73.3% vs. 60.1%, log-rank test, X^2^ = 10.006, P = 0.002, Figure 3C) and disease-free survival (DFS) (5-year survival rates, 67.5% vs. 53.4%, log-rank test, X^2^=9.502, P=0.001, Figure 3D) compared with low stromal PD-L2 expression. When the clinical stages were taken as stratifications, the associations were much significantly stronger for patients at early stages (stage I-II), between stromal PD-L2 expression and good OS (5-year survival rates, 92.5% vs. 60.1%, log-rank test, X2=14.219, P<0.001, Figure 4A) and DFS (5-year survival rates, 85.7% vs. 54.3%, log-rank test, X2 = 11.883, P=0.001, Figure 4B), while the associations were not significant for patients at the late stages (stage III-IV; Figure 4C and 4D). In contrast, high tumor PD-L2 expression was negatively correlated with disease-free survival (P=0.048, Figure 3E), but there was no significant correlation between the tumor PD-L2 expression level and overall survival time (P=0.134, Figure 3F). However, high tumor PD-L2 expression also had a poor DFS prognostic value at late stages (stage III-IV) (5-year survival rates, 45.8% vs. 56.9%, log-rank test, X2 = 5.001, P=0.025, Figure 4H), while it had no prognostic value for OS and early-stage patients (Figure 4E-G).

### Independency test of the association between PD-L2 expression and survival

Univariate Cox proportional hazard regression analysis showed that PD-L2 stromal expression (P=0.002), age (P=0.001) and well-known clinicopathologic prognostic parameters, such as T classification (P<0.001), N classification (P=0.001), recurrence or metastasis (P=0.005) and clinical stage (P<0.001), were significantly associated with survival (Table 3). Moreover, independency tests were carried out among the prognostic factors, including PD-L2 stromal expression and other clinicopathologic features (age, T classification, N classification, recurrence or metastasis and clinical stage), under a multivariate Cox proportional hazards regression model. In multivariate statistical analysis, we choose overall survival time as “time”, overall survival status as “status”, “death” as the reference (with a value of 1). We found that stromal PD-L2 was an independent and favorable factor for overall and cancer progression survival (OS hazards ratio: 0.722; 95% confidence interval: 0.528-0.986; P=0.041; DFS hazards ratio: 0.744; 95% confidence interval: 0.559-0.99; P=0.042) (Table 4). In addition, age, recurrence or metastasis and clinical stage were found to be independent prognostic predictors for overall survival and cancer progression survival. In contrast, tumor PD-L2 was an independent and worse factor for cancer progression survival (DFS hazards ratio: 1.423; 95% confidence interval: 1.033-1.961; P=0.031) (Table 4).

### Correlations of PD-L2 expression and CD68^+^ macrophages and CD4^+^Foxp3^+^ Treg cells in NPC tissue

To elucidate the roles of PD-L2 in immune regulation, we analyzed the correlation between the expression of stroma and tumor PD-L2 with the number of CD68^+^ macrophages and CD4^+^Foxp3^+^ Treg cells in the tumor and stroma, respectively, which have been implicated in the tumor microenvironment of previous reports 18-19. Among 557 NPC tissues, high stromal PD-L2 expression was positively correlated with the infiltration of CD68^+^ macrophages (51.52 ± 24.41/HPF vs. 42.33 ± 22.74/HPF, Pearson correlation=0.181; *P*<0.0001) and CD4^+^Foxp3^+^ Treg cells (7.49 ± 3.8/HPF vs. 6.96 ± 4.59/HPF, Pearson correlation=0.098; *P*=0.021; Table 5) in stromal tissue.

## Discussion

Immune checkpoint inhibitors are the main strategies of immunotherapy and have been widely used in various solid tumors. EBV-associated NPC is one of the most common head and neck malignancies, and its unique immune environment with massive T-cell infiltration provides a reasonable target for immunotherapy. However, the clinical benefit of immunotherapy is limited to a small number of patients with T-cell responses 20. To overcome the bottleneck of immunotherapy based on PD-1 and PD-L1 and screen immunotherapy markers for advanced NPC patients, we analyzed the prognostic value of intratumoral and interstitial immune indicators for patients with nasopharyngeal cancer.

Here, we found that overexpression of PD-L2 protein is likely correlated with NPC. PD-L2 protein was expressed in 90.8% of NPC tumor tissues and 80.8% of stromal tissues. Interestingly, we observed that distinct PD-L2 expression patterns correlated with different clinical outcomes. High stromal expression of PD-L2 predicted an improved survival rate, whereas high tumor abundance of PD-L2 was correlated with a poor disease-free survival rate. Furthermore, under a multivariate Cox proportional hazards regression model, stromal PD-L2 was an independent and favorable factor for overall and cancer progression survival, while tumor PD-L2 was an independent and worse factor for cancer progression survival. More importantly, further correlation analyses revealed that high stromal expression of PD-L2 in NPC was negatively associated with tumor T classification, recurrence and metastasis and advanced clinical stage, which suggested that stromal PD-L2 plays an antitumor role, whereas tumor PD-L2 may provide a selective advantage in NPC tumorigenic processes.

PD-L2 is the second PD-1 ligand discovered after PD-L1, and its immune regulation is complex and controversial. By binding to PD-1 on the surface of activated T cells, PD-L2 transmits negative signals to inhibit the proliferation of T cells and the secretion of cytokines, thereby inhibiting the immune function of T cells 21. Studies have found that strong PD-L2 expression in a variety of tumor tissues can play a negative role in tumor immune escape and predict poor overall survival, such as in liver cancer 22, esophageal cancer 23, prostate cancer 24, bladder cancer 25 and lung adenocarcinoma 26. Its high expression refers to tumor infiltration depth, lymph node and tumor metastasis 22. In this study, we also found that high levels of PD-L2 in NPC tumor tissues were correlated with unfavorable disease-free survival.

On the other hand, PD-L2 can bind to other receptors and play an immune costimulatory role. A study showed that PD-L2 binds repulsive guidance molecules B (RGMb) with the new type to form a supercomplex, initiates a series of downstream events such as downstream target gene transcription and cytoskeletal recombination, mediates T-cell amplification, and then plays an immune-activating role 27. In this study, we found that the high expression of PD-L2 in NPC stromal was contrary to that in nest, and there was a significant positive correlation between PD-L2 expression level and tumor-free survival time and overall survival time. Recent studies have found that, in addition to the PD-1 receptor, PD-L2 can also be combined with the unknown second receptor. Under the combined effects of anti-CD3 antibody subdose, PD-L2 stimulated T-cell proliferation more significantly than B7-1 to significantly enhance antitumor immune response, and this function is considered to be the main function of PD-L2 28-29. Previous experiments have shown that NPC patients with high expression of macrophages and associated regulatory T lymphocytes have a good prognosis 18-19. Here, we found that the high expression of PD-L2 in the stroma significantly increased the number of tumor-related CD68^+^ macrophages and CD4^+^Foxp3^+^ Treg regulatory T lymphocytes. Thus, the role of PD-L2 in stromal cell expression is opposite to that in tumor cells and plays a positive costimulatory effect in NPC stoma tissue.

The differential effects of PD-L2 on tumor cells and stromal cells may be due to different biological roles resulting from the binding of different receptors. Tumor cells may be combined with PD-1 to play an immune escape role and promote tumor progression; however, stromal cells may contribute to an antitumor immune response by associating with the unknown second receptor. Current research on PD-L2 is still in the initial stage, but the development of a study of PD-L2 and its various receptors will help further explore the effect and mechanism of the tumor immune microenvironment in tumor occurrence and development and provide a new set of therapeutic strategies.

## Supplementary Material

Supplementary figure and table.

## Figures and Tables

**Table 1:** The corresponding cutoff score of PD-L2 stroma and tumor expression for each clinicopathological feature according to ROC curve analysis

Feature	Cutoff score	*P* value
**PD-L2 stroma expression**		
T classification	4.5	0.001
N classification	1.5	0.120
Clinical stage	3.5	0.002
Cancer progression	2.5	0.001
Survival status	3.5	0.001
**PD-L2 tumor expression**		
T classification	4.5	0.625
N classification	4.5	0.962
Clinical stage	4.5	0.675
Cancer progression	5.5	0.198
Survival status	5.5	0.399

Note: PD-L2, programmed death ligand-2; ROC, receiver operating characteristic.

**Table 2:** Association of PD-L2 expression and clinicopathological characteristics in NPC patients

Characteristics	PD-L2 stroma staining		P value	Pearson's R	PD-L2 tumor staining		P value	Pearson's R
	low (n=193)	high (n=364)			low (n=430)	high (n=127)		
**Age, years**			0.011	-0.108			0.231	
<46	83	198			211	70		
≥46	110	166			219	57		
**Gender**			0.093				0.141	
Female	40	102			104	38		
Male	153	262			326	89		
**Tumor stage**			0.004	-0.123			0.501	
T1+T2	59	157			170	46		
T3+T4	134	207			260	81		
**Lymphoid Nodal (N) states**			0.258				0.947	
N0	45	101			113	33		
N1-3	148	263			317	94		
**Recurrence/metastasis**			0.003	-0.127			0.153	
No	113	236			282	67		
Yes	80	128			148	60		
**TNM clinical stage**			0.009	-0.111			0.564	
I+II	38	109			116	31		
III+IV	155	255			314	96		
**Histological type**			0.095				<0.001	0.171
NKUC	147	255			328	74		
NKDC	38	101			91	48		

Note: PD-L2, programmed death ligand-2; NKUC, non-keratinizing undifferentiated carcinoma; NKDC, non-keratinizing differentiated carcinoma; KSCC, keratinizing squamous cell carcinoma, 16 cases of patients diagnosed with KSCC were not statistically analyzed.

**Table 3:** Univariate Cox proportional hazards regression analysis for PD-L2

	5 year OS	95% CI	HR	*P* value	5 year DFS	95% CI	HR	*P* value
**Age**								
<46	75.4%	Reference			67.1%	Reference		
≥46	62.0%	1.261-2.316	1.709	0.001	58.0%	1.046-1.809	1.375	0.022
**Sex**								
Male	67.9%	Reference			60.8%	Reference		
Female	71.0%	0.626-1.268	0.891	0.52	68.1%	0.550-1.071	0.768	0.12
**Clinical stage**								
I+II	92.5%	Reference			85.7%	Reference		
III+IV	60.1%	3.535-11.993	6.511	<0.001	54.3%	2.532-6.245	3.976	<0.001
**Tumor stage**								
T1+T2	84.7%	Reference			78.7%	Reference		
T3+T4	58.5%	2.187-4.67	3.196	<0.001	52.4%	1.939-3.736	2.692	<0.001
**Lymph node (N) status**								
N0	80.1%	Reference			72.6%	Reference		
N1-3	64.6%	1.333-2.959	1.986	<0.001	59.0%	1.178-2.349	1.664	0.004
**Recurrence or metastasis status**								
Yes	28.6%	Reference			14.3%	Reference		
No	69.2%	1.357-8.047	3.305	0.008	63.1%	0.814-9.221	0.001	
**WHO histological classification**								
NKUC	69.8%	Reference			62.6%	Reference		
NKDC	67.6%	0.885-1.570	1.179	0.261	64.0%	0.918-2.083	1.302	0.885
**PD-L2 stroma expression**								
Low	60.1%	Reference			53.4%	Reference		
High	73.3%	0.459-0.836	0.620	0.002	67.5%	0.479-0.829	0.630	0.001
**PD-L2 tumor expression**								
Low	69.9%	Reference			64.6%	Reference		
High	64.6%	0.922-1.817	1.294	0.137	55.9%	1.000-1.846	1.359	0.048

Note: CI, confidence interval; WHO, World Health Organization; PD-L2, programmed death ligand-2; NKUC, non-keratinizing undifferentiated carcinoma; NKDC, non-keratinizing differentiated carcinoma; KSCC, keratinizing squamous cell carcinoma.

**Table 4:** Multivariate Cox proportional hazards regression analysis for PD-L2

	5 year OS	95% CI	HR	*P* value	5 year DFS	95% CI	HR	*P* value
**Age**								
<46	75.4%	Reference			66.4%	Reference		
≥46	62.0%	1.181-2.203	1.613	0.003	59.2%	1.006-1.762	1.332	0.045
**Clinical stage**								
I+II	92.5%	Reference			85.7%	Reference		
III+IV	60.1%	1.804-8.014	3.802	<0.001	54.3%	1.296-4.306	2.362	0.005
**Tumor stage**								
T1+T2	84.7%	Reference			78.7%	Reference		
T3+T4	58.5%	0.976-2.476	1.554	0.063	52.4%	1.000-2.374	1.541	0.050
**Lymph node (N) status**								
N0	80.1%	Reference			72.6%	Reference		
N1-3	64.6%	1.051-2.385	1.583	0.028	59%	0.986-2.007	1.406	0.060
**Recurrence or metastasis status**								
Yes	28.6%	Reference			14.3%	Reference		
No	69.2%	0.951-6.397	2.466	0.063	63.1%	1.205-6.623	2.825	0.017
**PD-L2 stroma expression**								
Low	60.1%	Reference			53.4%	Reference		
High	73.3%	0.528-0.986	0.722	0.041	67.5%	0.559-0.990	0.744	0.042
**PD-L2 tumor expression**								
Low	69.9%	Reference			64.6%	Reference		
High	64.6%	0.917-1.875	1.311	0.138	55.9%	1.033-1.961	1.423	0.031

Note: CI, confidence interval; PD-L2, programmed death ligand-2.

**Table 5:** Association between PD-L2 molecular expression and immune cell density

		CD68^+^ macrophagedensity in tumor	CD68^+^ macrophage density in stroma	CD4^+^Foxp3^+^ Treg density In stroma
PD-L2 tumor expression	Pearson Correlation	0.005	0.054	-0.007
	P value	0.902	0.207	0.871
PD-L2 stroma expression	Pearson Correlation	0.063	0.181	0.098
	P value	0.139	<0.0001	0.021
