# Supplementary material for: Expression of Programmed Death Ligand-2 is associated with Prognosis in Nasopharyngeal Carcinoma Microenviroment
Source: J Cancer. 2022 Nov 14;13(15):3606–14. doi: 10.7150/jca.77643 (PMC9809305; doi:10.7150/jca.77643)
Supplement: Supplementary file 1 — Supplementary figure and table. [file jcav13p3606s1.pdf]

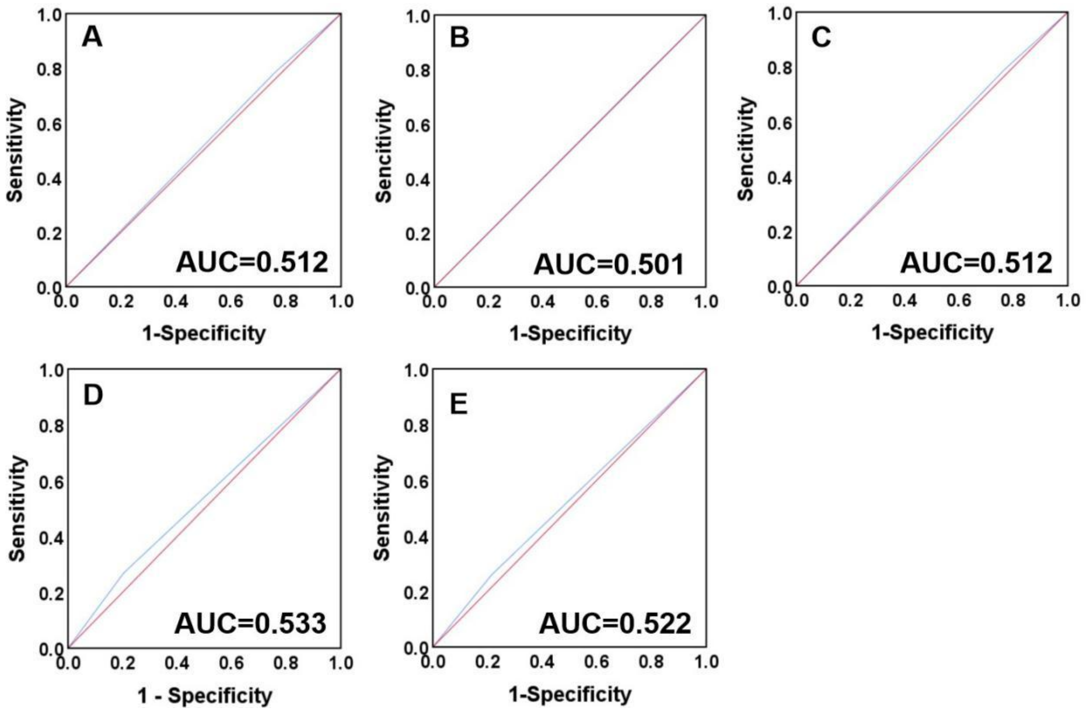

Supplemental Figure 1. Selection of the cutoff score. Receiver operating characteristic curve analysis was employed to determine the cutoff score for the designation of "high expression" of PD-L2. The sensitivity and specificity for each outcome of PD-L2 membranous staining of tumor tissues were plotted: A T classification (AUC=0.512, P =0.625); B N classification (AUC=0.501, P =0.962); C Clinical stage (AUC=0.512, P =0.675); D Cancer progression (AUC=0.533, P=0.198); E Survival status (AUC=0.522, P=0.399).

Supplemental Table 1. Characteristics of NPC patients

Characteristic	NPC patients(N=557)%
**Sex**	
Female	139(25.0)
Male	418(75.0)
**Age(years)**	
Median(range)	46(19-78)
Mean±SD	46.78±11.065
**Clinical stage**	
I-II	147(26.6)
III-IV	410(73.6)
**Progression**	
No	338(60.7)
Yes	219(39.3)
**Death**	
No	373(67.0)
Yes	184(33.0)
**Follow-up time(months)**	
Median(range)	67(2-99)
Mean±SD	58.63±1.097
**WHO histological classification**	
NKUC	402(72.2)
NKDC	139(25.0)
KSCC	16(2.8)

Note: SD, standard deviation; WHO, World Health Organization; NKUC, non-keratinizing undifferentiated carcinoma; NKDC, non-keratinizing differentiated carcinoma; KSCC, keratinizing squamous cell carcinoma.
